# Discrimination of cancerous from benign pigmented skin lesions based on multispectral autofluorescence lifetime imaging dermoscopy and machine learning

**DOI:** 10.1117/1.JBO.27.6.066002

**Published:** 2022-06-14

**Authors:** Priyanka Vasanthakumari, Renan A. Romano, Ramon G. T. Rosa, Ana G. Salvio, Vladislav Yakovlev, Cristina Kurachi, Jason M. Hirshburg, Javier A. Jo

**Affiliations:** a Texas A&M University, Department of Biomedical Engineering, College Station, Texas, United States; b University of São Paulo, São Carlos Institute of Physics, São Paulo, Brazil; c Skin Department of Amaral Carvalho Hospital, São Paulo, Brazil; d University of Oklahoma Health Science Center, Department of Dermatology, Oklahoma City, Oklahoma, United States; e University of Oklahoma, School of Electrical and Computer Engineering, Norman, Oklahoma, United States

**Keywords:** skin cancer, autofluorescence, fluorescence lifetime imaging, feature selection, machine learning, computer-aided diagnosis

## Abstract

**Significance:**

Accurate early diagnosis of malignant skin lesions is critical in providing adequate and timely treatment; unfortunately, initial clinical evaluation of similar-looking benign and malignant skin lesions can result in missed diagnosis of malignant lesions and unnecessary biopsy of benign ones.

**Aim:**

To develop and validate a label-free and objective image-guided strategy for the clinical evaluation of suspicious pigmented skin lesions based on multispectral autofluorescence lifetime imaging (maFLIM) dermoscopy.

**Approach:**

We tested the hypothesis that maFLIM-derived autofluorescence global features can be used in machine-learning (ML) models to discriminate malignant from benign pigmented skin lesions. Clinical widefield maFLIM dermoscopy imaging of 41 benign and 19 malignant pigmented skin lesions from 30 patients were acquired prior to tissue biopsy sampling. Three different pools of global image-level maFLIM features were extracted: multispectral intensity, time-domain biexponential, and frequency-domain phasor features. The classification potential of each feature pool to discriminate benign versus malignant pigmented skin lesions was evaluated by training quadratic discriminant analysis (QDA) classification models and applying a leave-one-patient-out cross-validation strategy.

**Results:**

Classification performance estimates obtained after unbiased feature selection were as follows: 68% sensitivity and 80% specificity with the phasor feature pool, 84% sensitivity, and 71% specificity with the biexponential feature pool, and 84% sensitivity and 32% specificity with the intensity feature pool. Ensemble combinations of QDA models trained with phasor and biexponential features yielded sensitivity of 84% and specificity of 90%, outperforming all other models considered.

**Conclusions:**

Simple classification ML models based on time-resolved (biexponential and phasor) autofluorescence global features extracted from maFLIM dermoscopy images have the potential to provide objective discrimination of malignant from benign pigmented lesions. ML-assisted maFLIM dermoscopy could potentially assist with the clinical evaluation of suspicious lesions and the identification of those patients benefiting the most from biopsy examination.

## 1 Introduction

Skin cancer is the most common type of cancer in the United States, with melanoma being the fifth most prevalent among men and women.^1^ The 5-year survival rate of patients with early-stage skin melanoma is ∼94%; however, ∼13% of skin melanoma patients are diagnosed with lesions already at intermediate or advance stages,^1^ which are associated with 5-year survival rates of ∼61% and ∼27%, respectively. The most common diagnosis strategy for skin cancer is clinical evaluation of suspicious lesions followed by biopsy for histopathological evaluation to confirm diagnosis and tissue staging. One major drawback of this practice is the inability to clinically distinguish between similar lesions; in particular, melanoma is often mistaken for other benign pigmented lesions such as seborrheic keratosis (pSK). In addition, it is known that the accuracy of melanoma diagnosis with unaided eye is only about 60%.^2^ Therefore, clinical tools that could provide objective, *in situ,* and accurate noninvasive discrimination between malignant and benign skin lesions during clinical examination could significantly improve early detection of skin cancer, reduce the risk of adverse events, and lead to improved cost-conscious patient care.

One of the most common tools used by physicians to diagnose skin cancer lesions is the dermoscope^2^^–^^4^ which helps the unaided eye by magnifying the features on the skin. This allows doctors to examine the morphological features of concerning lesions at a significantly more detailed level. Although dermoscopy is known to improve the diagnostic sensitivity of skin lesions by ∼10% to 30%, its performance largely depends on both the level of experience of the dermatologist and the type of lesions.^2^ The highly subjective nature and poor reproducibility of this method have led to the emergence of several proposed computer-aided diagnostic (CAD) systems.^3^^,^^5^^–^^7^

CAD systems are becoming largely popular in both diagnosis and prognosis of various diseases as they allow automated and noninvasive analysis of the tissue conditions. Table 1 summarizes some of the published works that reports the diagnosis and classification of pigmented skin lesions. Most of the works used dermoscopic images that were either collected by the authors or from publicly available datasets (e.g., ISIC archive, ISBI, Atlas, HAM10000, or PH2). Celebi and Zornberg^8^ explored the clinically significant colors in dermoscopic images using K-means clustering and employed symbolic regression to classify the lesions. Ramlakhan and Shang^9^ designed a melanoma recognition system using smart phone photographs that are classified using k-nearest neighbor (kNN) algorithm. Satheesha et al.^10^ examined computerized three-dimensional (3D) dermoscopy features of skin cancer lesions to develop multiclass classifiers using Adaboost, bag of features (BoF), and support vector machine (SVM) techniques. Khristoforova et al.^11^ used logistic regression to classify benign and malignant skin lesions using spectral features from Raman and autofluorescence spectroscopy measurements.

**Table 1:** Summary of previously reported works on pigmented skin lesion classification.

Imaging modality	Distribution of patients or images	Classification task	Algorithm	Performance	Validation or testing technique	Reference
Dermoscopy	Total images: 914 Melanoma: 272 Blue nevi: 28 Dysplastic nevi: 405 Congenital nevi: 17 Dermal nevi: 33 Dermatofibroma: 20 Reed/Spitz nevi: 79 Seborrheic keratoses: 47	Benign versus malignant	K-means clustering and symbolic regression	Sensitivity: 62% Specificity: 76%	Train-test sets	Celebi and Zornberg^8^
Smart phone photograph	Benign: 37 Malignant: 46	Benign versus malignant	kNN	Accuracy: 66.7% Sensitivity: 60.7% Specificity: 80.5%	Train-test sets	Ramlakhan and Shang^9^
Computerized dermoscopy (3D)	PH2 dataset Total images: 200 Melanoma *In situ* melanoma Atypical nevus Common nevus	Multiclass classifier (PH2: 4 classes and ATLAS: 8 classes)	SVM, AdaBoost, BoF	PH2 dataset Sensitivity: 96% Specificity: 97%	Leave-one-out cross-validation	Satheesha et al.^10^
	ATLAS dataset Total images: 63 Melanoma *In situ* melanoma BCC Blue nevus Dermatofibroma Haemangioma pSK Normal mole			ATLAS dataset Sensitivity: 98% Specificity: 99%		
Raman and autofluorescence spectroscopy	Total patients: 56 Melanoma: 19 BCC: 18 Benign: 19	Benign versus malignant	Binary logistic regression	Accuracy: 87% Sensitivity: 84% Specificity: 89%	No independent validation	Khristoforova et al.^11^
FLIM	Melanoma: 43 BCC: 28 SCC: 67	Early-stage cancer versus advanced-stage cancer	RF, kNN, SVM, LDA	Accuracy: 84.62% AUC: 1	Bootstrapping	Yang et al.^12^
Dermoscopy	Total images: 2000 Nevus: 1372 Melanoma: 374 Seborrheic keratosis: 254	Three classes: nevus, melanoma, seborrheic keratosis	Ensemble of CNNs	AUC: 0.891	Training, validation, and test sets	Harangi^13^
Dermoscopy	ISBI 2016 database Training images: 900 Test images: 379	Benign versus malignant	CNN	Accuracy: 81.33% Sensitivity: 78.6% Precision: 79.74%	Train-test sets	Romero Lopez et al.^14^
Dermoscopy	ISIC database Training images: 900 Benign: 727 Melanoma: 173 Test images: 379 Benign: 304 Melanoma: 75	Benign versus malignant	CNN + SVM	Accuracy: 80.5% Sensitivity: 53.3% Specificity: 87.2%	Train-test sets	Majtner et al.^15^
Dermoscopy	HAM10000 dataset Training images: 10,015 Validation images: 193 Test images: 1512	Seven classes: melanoma, melanocytic nevus, BCC, actinic keratosis, Bowens disease, benign keratosis, dermatofibroma, vascular lesion	WonDerm Fine-tuned neural networks (Ensemble)	Validation accuracy: 89.9% Test accuracy: 78.5%	Training, validation, and test sets	Lee et al.^16^
Dermoscopy	ISBI and PH2 datasets Benign: 3319 Melanoma: 830	Benign versus melanoma	Feature extraction using AlexNet and VGG16 Classification: ensemble of kNN, discriminant analysis, SVM, and tree	Accuracy: 99.0% Sensitivity: 99.52% Specificity: 98.59%	Fivefold CV and 0.5 hold out CV	Amin et al.^17^
Dermoscopy	ISIC dataset Malignant: 117 Benign: 481	Benign versus malignant	CNN: ResNet 152	Accuracy: 90.4% Sensitivity: 82.0% Specificity: 92.5%	Train-test sets	JoJoa Acosta et al.^18^

CNN, convolutional neural network; RF, random forests; LDA, linear discriminant analysis; SVM, support vector machine; kNN, k-nearest neighbor; FLIM, fluorescence lifetime imaging; AUC, area under the curve; CV, cross-validation; BCC, basal cell carcinoma.

Classification of dermoscopy images of benign and malignant skin lesions using different deep learning approaches has also been reported. Harangi^13^ used an ensemble of different convolutional neural network (CNN) classifiers, while Romero Lopez et al.^14^ used transfer learning with pretrained VGGNet CNN architecture. Majtner et al.^15^ combined CNN with SVM classifier using handcrafted RSurf features and local binary patterns to classify melanomas from other benign skin lesions. Lee et al.^16^ developed the WonDerm pipeline that segments the skin cancer dermoscopic images using neural network architectures and classifies it using an ensemble approach. Amin et al.^17^ extracted features using pretrained AlexNet and VGG16 deep learning architectures, performed feature selection using principal component analysis, and applied traditional machine learning models including SVM, kNN, and discriminant analysis. Jojoa Acosta et al.^18^ utilized transfer learning with ResNet-152 architecture to classify benign and malignant skin lesions using dermoscopic images.

It has been widely established that autofluorescence responses of intrinsic fluorophores vary significantly between normal and neoplastic tissues.^19^^–^^23^ Neoplastic progressions in the epithelial tissue are associated with morphological, biochemical, and functional alterations which can cause changes in the autofluorescence responses from the tissue.^22^^–^^24^ The skin has several intrinsic fluorophores, such as nicotinamide adenine dinucleotide (NADH), flavin adenine dinucleotide (FAD), collagen, elastin, keratin, melanin, and porphyrins.^19^^,^^25^^–^^27^ The levels of two metabolic cofactors and endogenous fluorophores in the epidermis, the reduced-form NADH and FAD, can change as skin cancer develops.^19^^,^^24^ The optical redox ratio, typically defined as the ratio of fluorescence intensity of NADH to FAD, is sensitive to changes in the cellular metabolic rate. Increased cellular metabolic activity, a hallmark of neoplastic cell transformation, is usually attributed to a decrease in the optical redox ratio. In addition, the fluorescence lifetimes of these metabolic cofactors are sensitive to protein binding, thus to cellular metabolic pathways involving NADH and FAD. As a result, carcinogenesis process has been shown to cause changes in both NADH and FAD fluorescence lifetimes. Finally, cancer development also leads to extracellular matrix remodeling occurring within the dermis, which together with concurring epidermis thickening, result in a decrease in connective tissue autofluorescence that can be measured. Therefore, interrogation of NADH, FAD, and collagen autofluorescence could provide optical biomarkers of skin epithelial cancer.

Preferential excitation of these endogenous fluorophores in the tissue by multiple excitation sources could shed light on the biochemical changes in the target lesion area.^28^ The broad emission spectral bandwidth of the fluorescence intensity signal has an intrinsic disadvantage in that it is difficult to differentiate between the intensities of overlapping emissions from multiple fluorophores. Time-resolved technique such as multispectral autofluorescence lifetime imaging (maFLIM) overcomes this challenge by quantifying the fluorescence lifetime in addition to the emission spectrum. Alex et al.^29^ demonstrated fluorescence lifetime-based imaging of minipig skin and human skin to specifically target the endogenous fluorophores: keratin, NADH, melanin, elastin, and collagen under 725-nm multiphoton excitation. The capability of such optical biopsy techniques to serve as promising tools for dermatological research to facilitate preclinical and clinical translation is also highlighted. Huck et al.^30^ demonstrated the effectiveness of the combined modality, multiphoton-based intravital tomography and fluorescence lifetime imaging to monitor the progression of inflammatory skin diseases. The two modalities studied the biochemical changes induced by the redistribution of mitochondria at different stages of inflammatory skin conditions.

Several animal and human tissue studies have been published on the autofluorescence properties of skin cancer lesions.^31^^–^^35^ Pastore et al.^36^ conducted experiments with mouse models to study the autofluorescence response from melanoma skin lesions using multiphoton excitation at 740 and 900 nm and emission spectral bands at 447 and 540 nm. A significant difference in the bound and free NADH ratio between cancerous and noncancerous sites was observed, while the fluorescence decay obtained from targeting FAD remained almost the same between the two regions. It was also mentioned that the presence of melanin in the deeper layers of the skin tissue could interfere with the overall fluorescence response from the lesions. Miller et al.^32^ studied the autofluorescence emission properties between squamous cell carcinoma (SCC) bearing and normal mice skin under 480-nm excitation, and a decrease in the short lifetime component for SCC in comparison to normal skin was observed for 535-nm emission band. Drakaki et al.^37^ studied the autofluorescence responses from mouse, chicken, and pig skins under ultraviolet (UV) excitation, and the structural differences and the variations in tissue constituents were investigated between the different animal species for an emission spectral band between 340 and 950 nm. De Beule et al.^34^ investigated the autofluorescence response from *ex vivo* biopsy skin lesions under 355- and 440-nm excitations, and the average fluorescence lifetime was found to be useful in discriminating basal cell carcinoma (BCC) from normal skin tissues at the emission band between 390 and 600 nm. Galletly et al.^31^ imaged unstained human biopsy samples using maFLIM under a 355-nm pulsed laser excitation, and significant differences in the mean fluorescence lifetimes for the emission wavelengths 375 and 455 nm were observed between the autofluorescence responses from BCC skin lesions and healthy skin. Lohmann and Bodeker^38^ analyzed the fluorescence intensities at the emission wavelength 470 nm, from human skin with melanoma, nevi, and dysplastic nevi lesions under 365-nm excitation, and a significant difference in fluorescence intensities was observed for melanoma and nevi lesions, while melanoma and dysplastic nevi lesions did not show much difference. Fast et al.^39^ investigated the autofluorescence response from human skin at 780-nm frequency doubled excitation and two emission channels at 535 and 720 nm corresponding to red and green channels. Red channel collects fluorescence emission from melanin, while the green channel collects emission from keratin, NAD(P)H, FAD, and elastin.

In this work, we developed and validated a label-free and objective image-guided strategy for the clinical evaluation of suspicious pigmented skin lesions based on maFLIM dermoscopy. In addition, a computationally efficient frequency-domain deconvolution of maFLIM data is explored, and three different pools of global image-level maFLIM features were evaluated for machine-learning (ML)-based objective discrimination between malignant and benign pigmented skin lesions.

## 2 Methods

**Fig. 1:**
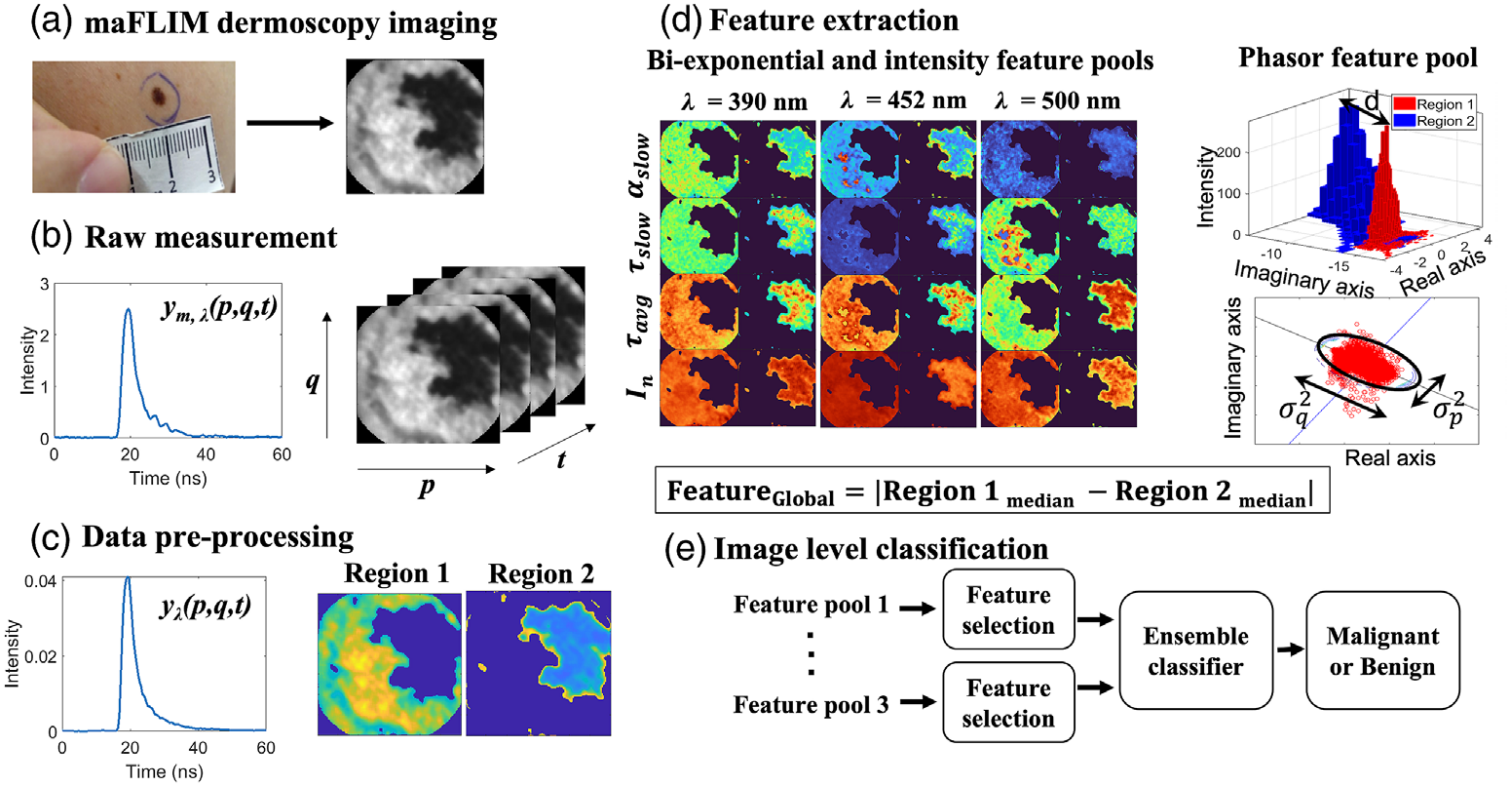
Summary of methodology showing maFLIM image acquisition, preprocessing, feature extraction, and classification. maFLIM, multispectral autofluorescence lifetime imaging.

A summary of the complete methodology performed in this study is shown in Fig. 1.

### 2.1 maFLIM Dermoscopy Imaging of Skin Lesions

A total of 30 patients ($n_{\text{patients}}=30$) from the Dermatology Department of the Amaral Carvalho Cancer Hospital (Jahu, Sao Paulo, Brazil) were recruited for this study, following a human study protocol approved by the Internal Review Board of that institution (CAAE: 71208817.5.00005434). Only patients presenting at least one pigmented skin lesion undergoing biopsy examination for skin cancer diagnosis were recruited. The pigmented skin lesions considered in this work are solar lentigo, pSK, pigmented superficial BCC, pigmented nodular BCC, and melanoma.

maFLIM images were obtained from clinically suspicious lesions using an in-house developed time-domain maFLIM dermoscope previously described.^40^ With this maFLIM dermoscope, skin tissue autofluorescence is simultaneously imaged at three emission bands (390±20, 452±22.5, and >496  nm, preferentially targeting collagen, NADH, and FAD autofluorescence emission, respectively) with a temporal resolution of 0.4 ns, field-of-view (FOV) of 8.65 ⋅ $8.65\;{\mathrm{mm}}^{2}$, and lateral resolution of 120  μm. For the rest of the paper, the emission wavelengths at the three spectral channels will be more conveniently referred to as 390, 452, and 500 nm. After signing the corresponding written informed consent form, each patient underwent the following imaging protocol right before the scheduled biopsy examination procedure. First, the lesion was gently cleaned with a gauze soaked in a saline solution. Then, the tip of the maFLIM dermoscope, previously disinfected using a gauze soaked in 70% ethanol, was placed in contact with the lesion, and an maFLIM image was acquired. The imaging site was selected so regions within and outside the visible lesion were present within the FOV of the maFLIM dermoscope. Right after maFLIM imaging, lesion tissue biopsy was performed following standard procedures. Each maFLIM image was labeled based on the histopathological evaluation of the lesion biopsy, which was considered the gold standard in this study. All images were acquired with a laser excitation at 355 nm and average excitation power of 10 mW measured at the sample, 140×140  pixels per image, and at a pixel rate of 10 kHz. These image acquisition parameters corresponded to an acquisition time of 1.96 s per image and an excitation energy exposure of 1.96 mJ at the sample, which is significantly lower than the maximum permissible exposure levels for skin based on guidelines from the American National Standards Institute – ANSI.^41^ The total number of lesions imaged from the 30 patients was 60 (i.e., $n_{\text{lesions}}=60$). An instrument response function (IRF) was measured by acquiring the reflection of excitation pulse by placing a mirror at the sample end.

### 2.2 maFLIM Data Preprocessing

**Fig. 2:**
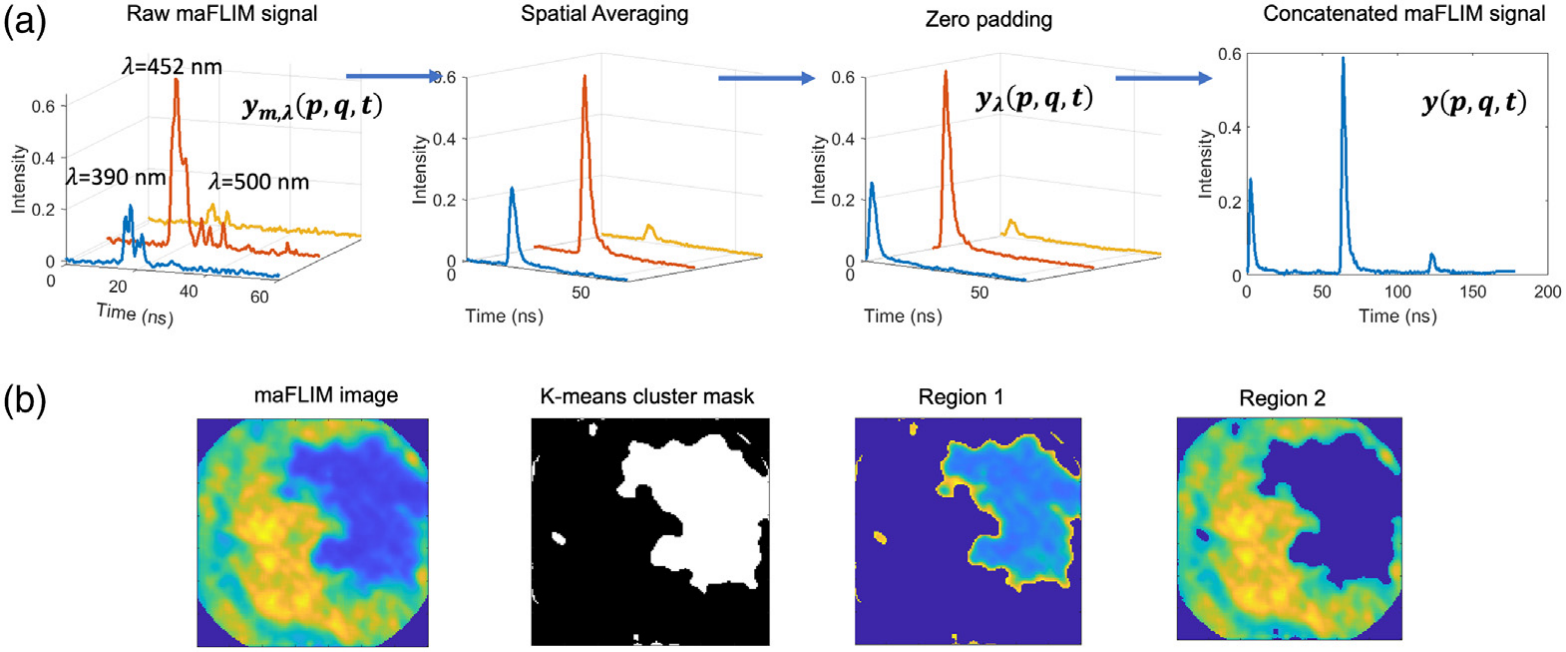
(a) Transformations in a single pixel multispectral maFLIM data during pixel-level preprocessing. (b) Example maFLIM image with K-means cluster mask and the two separated regions. The images map the total integrated intensity of the maFLIM signals at each pixel location. maFLIM, multispectral autofluorescence lifetime imaging.

Pixel-level preprocessing: The maFLIM data measured at each image pixel (p,q) are composed of three fluorescence intensity temporal decay signals $y_{m,\lambda }(p,q,t)$ measured at the three targeted emission spectral bands (λ). The preprocessing steps applied to each pixel maFLIM temporal signal is shown in Fig. 2(a). First, offset subtraction was applied to the raw maFLIM signal, $y_{m,\lambda }(p,q,t)$, followed by spatial averaging (order 5×5) to increase the signal-to-noise ratio (SNR) of the time-dependent signal. The offset was estimated by fitting a straight line on the first and last five time points in each channel. The baseline was then subtracted from the entire time vector to obtain the corrected signal. Since the background fluorescence was significantly lower than the sample fluorescence, the background correction of the signals was not performed. Second, the duration of the temporal decay signals for all emission bands was adjusted to the length of the longest signal among the three emission channels, which is 149 temporal samples (59.6 ns) by applying zero padding to the short signals. Finally, the signals from the three emission channels, $y_{\lambda }(p,q,t)$, are concatenated to form y(p,q,t) as shown in Eq. (1). Signal concatenation is essential for cluster analysis in image level preprocessing, explained later in this section, as well as for frequency-domain deconvolution explained in Sec. 2.3.2. The concatenated signal at each pixel location can be represented as

$$y(p,q,t)=\overset{2}{\underset{n=0}{\sum }}y_{\lambda_{n+1}}(p,q,(t-M.n)),$$ (1)

where y(p,q,t) is the preprocessed concatenated maFLIM decay signal; $y_{\lambda_{1}}(p,q,t)$, $y_{\lambda_{2}}(p,q,t)$, and $y_{\lambda_{3}}(p,q,t)$ are the preprocessed maFLIM decay signals from each of the three spectral channels, $\lambda_{1}=390\;\mathrm{nm}$, $\lambda_{2}=452\;\mathrm{nm}$, and $\lambda_{3}=500\;\mathrm{nm}$; M is the temporal spacing between the signals from the three channels; (p,q) indicates the pixel locations. The value of M is equal to 149, which is the length of the fluorescence emission decays in each channel.

Image-level preprocessing: Pixels presenting either signal saturation or low SNR (<15  dB) were detected and masked. The majority of the acquired maFLIM images contain pixels from within and outside the skin lesion region; thus, cluster analysis was performed to group pixels based on their region of origin. The concatenated signal y(p,q,t) is used as the feature vector for cluster analysis at each pixel location to simultaneously include the information from the three emission channels. The steps for cluster analysis are as follows: First, an unsupervised K-means clustering algorithm was applied to generate two cluster masks. Then, each cluster mask is applied to the maFLIM image to define two regions within the FOV of the maFLIM image. It should be noted that since the K-means clustering algorithm involves random initialization of cluster centroids, it is difficult to identify which cluster mask belongs to within or outside the skin lesion region; thus, the identified regions were taken as two arbitrary regions: region-1 and region-2. Figure 2(b) shows an example of the cluster mask and the two separated regions generated from a representative maFLIM image.

### 2.3 Feature Extraction

#### 2.3.1 Features based on time-domain deconvolution parameter estimation

In the context of time-domain maFLIM data analysis, the fluorescence decay $y_{\lambda }(p,q,t)$ measured at each emission spectral band (λ) and spatial location (p,q) can be modeled^42^ as the convolution of the fluorescence impulse response (FIR) $h_{\lambda }(p,q,t)$ of the sample and the measured IRF $u_{\lambda }(t)$:

$$y_{\lambda }(p,q,t)=u_{\lambda }(t)*h_{\lambda }(p,q,t).$$ (2)

The standard method for time-domain maFLIM data analysis proceeds by first deconvolving the IRF of each spectral band ($u_{\lambda }(t)$) from the corresponding measured time-resolved fluorescence signal $y_{\lambda }(p,q,t)$ to estimate the sample FIR for each image pixel, $h_{\lambda }(p,q,t$),^39^ which is usually modeled as a multiexponential decay. The model order (number of exponential components) can be selected by analyzing the model-fitting mean squares error (MSE) as a function of the model order. For the maFLIM data of this study, a model order of two was selected, since the addition of a third component did not reduce the MSE. The variations in error for one, two, and three exponential components during fitting is shown in Fig. S1 in the Supplemental Material. The FIR was modeled as

$$h_{\lambda }(p,q,t)=\alpha_{\text{fast},\lambda }e^{\frac{-t}{\tau_{\text{fast},\lambda }(p,q)}}+\alpha_{\text{slow},\lambda }e^{\frac{-t}{\tau_{\text{slow},\lambda }(p,q)}},$$ (3)

where $\tau_{\text{fast},\lambda }$ and $\tau_{\text{slow},\lambda }$ represent the time-constant (lifetime) of the fast and slow decay components, respectively; while $\alpha_{\text{fast},\lambda }$ and $\alpha_{\text{slow},\lambda }$ represent the contribution of the fast and slow decay components, respectively. The average fluorescence lifetime for each spectral band at each pixel location is computed as

$$\tau_{\mathrm{avg},\lambda }(p,q)=\frac{\int th_{\lambda }(p,q,t)dt}{\int h_{\lambda }(p,q,t)dt}.$$ (4)

The parameters of the biexponential decay model are estimated for each pixel by nonlinear least squares iterative reconvolution.^42^ After deconvolution, the biexponential parameters estimated at each pixel can be used as features representing the temporal dynamics of the fluorescence decays at each emission spectral band: $\alpha_{\text{fast},\lambda }(p,q)$, $\alpha_{\text{slow},\lambda }(p,q)$, $\tau_{\text{fast},\lambda }(p,q)$, $\tau_{\text{slow},\lambda }(p,q)$, and $\tau_{\mathrm{avg},\lambda }(p,q)$. Since the sum of $\alpha_{\text{fast},\lambda }(p,q)$ and $\alpha_{\text{slow},\lambda }(p,q)$ is equal to one, only one of them is kept as a feature.

In addition, the following spectral intensity features can be also estimated from the deconvolved FIR, $h_{\lambda }(p,q,t)$. Absolute fluorescence intensities $I_{\lambda }(p,q)$ for each emission spectral bands are simply computed by time integrating the FIR $h_{\lambda }(p,q,t)$:

$$I_{\lambda }(p,q)=\int h_{\lambda }(p,q,t)dt.$$ (5)

The normalized fluorescence intensities $I_{\lambda ,n}(p,q)$ can be also computed from the multispectral absolute fluorescence intensities $I_{\lambda }(p,q)$ as follows:

$$I_{\lambda ,n}(p,q)=\frac{I_{\lambda }(p,q)}{\sum_{\lambda }I_{\lambda }(p,q)}.$$ (6)

Lastly, the ratio of absolute intensities from the three spectral channels is computed at each pixel location resulting in three additional features:

$$I_{390,n}/I_{452,n}(p,q)=\frac{I_{390,n}(p,q)}{I_{452,n}(p,q)},$$ (7)

$$I_{452,n}/I_{500,n}(p,q)=\frac{I_{452,n}(p,q)}{I_{500,n}(p,q)},$$ (8)

$$I_{390,n}/I_{500,n}(p,q)=\frac{I_{390,n}(p,q)}{I_{500,n}(p,q)}.$$ (9)

In general, the features can be computed at the pixel or the image level. In this study, image-level global features were explored, whereby one set of features, a single feature vector, is estimated to represent the whole image. Each global feature was computed from the corresponding pixel-level maFLIM feature map as follows. Based on the two regions identified per image using the cluster analysis described in Sec. 2.2, the feature median value for each region was computed, and the absolute value of their difference was taken as the global feature:

$${\text{Feature}}_{\text{Global}}=|\text{median}({\text{Feature}}_{\text{pixel}-\text{level},\text{Region}-1})-\text{median}({\text{Feature}}_{\text{pixel}-\text{level},\text{Region}-2})|.$$ (10)

Since ${\mathrm{Feature}}_{\text{Global}}$ is the absolute difference between the feature median values from the two clustered regions, it is independent of labeling the regions as either lesion or healthy. This is particularly beneficial as it is difficult to label the clustered regions due to the unavailability of the pixel-level ground truth labels as well as the randomness in the K-means algorithm. These defined global features have the advantage of reducing patient-to-patient variability in the extracted features, which is particularly important as the color and texture of skin vary considerably with ethnicity and age. This feature extraction approach based on time-domain deconvolution of the maFLIM data generates a total of six intensity and 12 biexponential global maFLIM features, as summarized in Table 2.

**Table 2:** Feature set showing both intensity and biexponential global maFLIM features.

Intensity features		Biexponential features		
$I_{390,n}$	$I_{390,n}/I_{452,n}$	$\alpha_{\text{fast},390}$	$\alpha_{\text{fast},452}$	$\alpha_{\text{fast},500}$
$I_{452,n}$	$I_{452,n}/I_{500,n}$	$\tau_{\text{slow},390}$	$\tau_{\text{slow},452}$	$\tau_{\text{slow},500}$
$I_{500,n}$	$I_{390,n}/I_{500,n}$	$\tau_{\text{fast},390}$	$\tau_{\text{fast},452}$	$\tau_{\text{fast},500}$
—	—	$\tau_{\mathrm{avg},390}$	$\tau_{\mathrm{avg},452}$	$\tau_{\mathrm{avg},500}$

#### 2.3.2 Phasor-based features from frequency-domain deconvolved signals

As mentioned in Sec. 2.3.1, the parameters of the biexponential decay model are estimated for each pixel by nonlinear least squares iterative reconvolution, which is computationally expensive and time consuming. This brings about the need to develop a much simpler algorithm for extracting maFLIM features with comparatively similar discriminative capability. An alternate fitting-free strategy explored in this work is inspired by Campos-Delgado et al.,^43^ where a model-free representation of maFLIM data was developed utilizing the frequency-domain properties of the phasor representations. Here, we aim to replace the iterative time-domain deconvolution process by a simple division operation in the frequency domain.^44^ The computational overload is further reduced by processing the fluorescence decays of all the three spectral channels together, unlike in the traditional method where the maFLIM signal for each spectral channel must be processed separately. Subsequently, several features can be extracted from the frequency-domain phasor representation of the maFLIM data.^45^^,^^46^ The presented method proceeds in three steps: (1) performing frequency-domain deconvolution of the instrument response from the concatenated pre-processed fluorescence decays from all the three spectral channels, (2) constructing phasor plots for the maFLIM data, and (3) extracting global features from the phasor plots representing each maFLIM image. A detailed description of this method is presented as follows.

In this approach, the preprocessed and concatenated maFLIM signals at each pixel location, y(p,q,t) are normalized to sum one for further processing and feature extraction. Similar to the concatenated, preprocessed signal, y(p,q,t), the concatenated IRF from all the three spectral channels can be mathematically represented as

$$u(t)=\overset{2}{\underset{n=0}{\sum }}u_{\lambda_{n+1}}(t-M.n),$$ (11)

where u(t) is the concatenated IRF; $u_{\lambda_{1}}(t)$, $\;u_{\lambda_{2}}(t)$, and $u_{\lambda_{3}}(t)$ are the IRF signals from the three spectral channels; M is the temporal spacing between the signals from the three channels; and (p,q) indicates the pixel positions.

The first step of the algorithm is to compute the Fourier transform (FT) of both the preprocessed concatenated signal and the concatenated IRF. The FT of the signals y(p,q,t) and u(t) can be represented as Y(p,q,ω) and U(ω), respectively, where ω is the angular frequency:

Y(p,q,ω)=FT{y(p,q,t)}, (12)

U(ω)=FT{u(t)}. (13)

If the effective FIR from all the three fluorescence emission channels is denoted as h(p,q,t), the effective fluorescence frequency response H(p,q,ω) is obtained from the FT of h(p,q,t) as

H(p,q,ω)=FT{h(p,q,t)}. (14)

Therefore, the convolution in Eq. (1) can be represented as a multiplication in the frequency domain as

Y(p,q,ω)=H(p,q,ω)U(ω). (15)

To uniformly scale all the frequency components of Y(p,q,ω), the normalization with respect to the DC response Y(p,q,0) can be applied as follows:

$$\frac{Y(p,q,\omega )}{Y(p,q,0)}=\frac{H(p,q,\omega )U(\omega )}{H(p,q,0)U(0)}.$$ (16)

Subsequently, the normalized fluorescence frequency response P(p,q,ω) can be estimated as

$$P(p,q,\omega )=\frac{H(p,q,\omega )}{H(p,q,0)}=\frac{Y(p,q,\omega )U(0)}{U(\omega )Y(p,q,0)}.$$ (17)

**Fig. 3:**
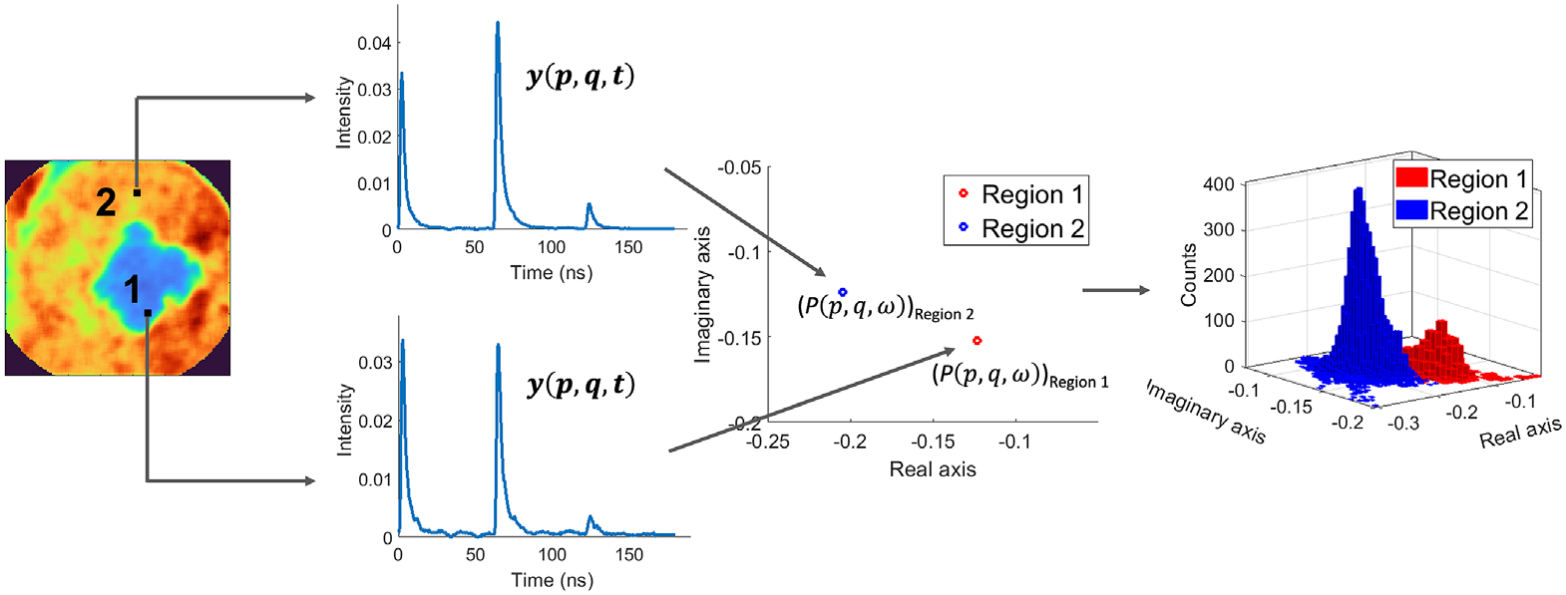
Transition of a sample maFLIM image to the corresponding 2D histogram distribution on the phasor plot. Figure also shows the transformation of pixels from both regions 1 and 2 on the maFLIM image into corresponding points on the phasor plot computed at an arbitrary frequency component.

A phasor representation for the normalized frequency response P(p,q,ω) at specific values of the frequency ω can be generated by plotting the real Re[P(p,q,ω)] versus the imaginary Im[P(p,q,ω)] components of P(p,q,ω). Therefore, each pixel of the maFLIM image is mapped to a point in the corresponding phasor plot generated for a specific frequency. This transformation is shown in Fig. 3, where a representative maFLIM image is mapped to its corresponding phasor plot for an arbitrary frequency. Region-1 and region-2 marked on the maFLIM image represent the regions obtained after clustering. The pixels of each region are mapped into a two-dimensional (2D)-histogram distribution on the phasor plot, as shown in Fig. 3.

**Fig. 4:**
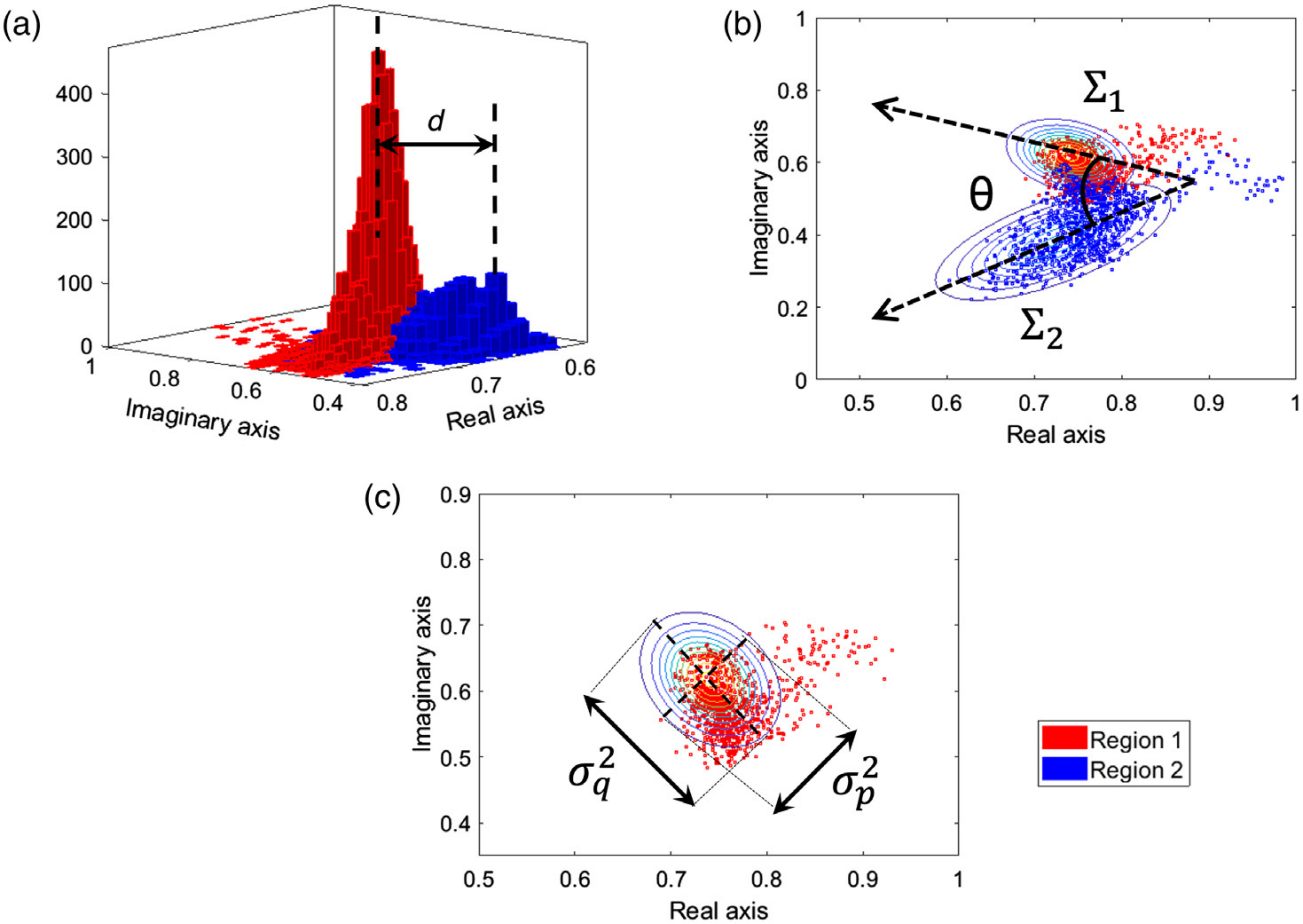
(a) 2D histogram phasor distributions from the pixels corresponding to the two regions in an maFLIM image. The distance between the distributions is indicated by “d.” (b) Phasor distribution scatter plots with bivariate Gaussian fits on regions 1 and 2. The covariance matrices $\Sigma_{1}$ and $\Sigma_{2}$ give a measure of spread of the two regions and θ represents the angle between their major axes. (c) Phasor distribution scatter plot showing the variances $\sigma_{p}^{2}$ and $\sigma_{q}^{2}$ along the major axes. The ratio of the variances indicates the symmetry of the distribution.

From the phasor representations of the maFLIM images at specific frequency components, ω=2πf, the following features were computed as follows. First, a bivariate Gaussian function was fitted to the phasor distribution of each region (region-1 and region-2) of a given maFLIM image: $f_{1}=N(\mu_{1},\Sigma_{1})$, $f_{2}=N(\mu_{2},\Sigma_{2})$ (Fig. 4). The “distance” between the phasor distributions of the two regions was then estimated as the magnitude of the difference of the distribution means: $d=|\mu_{1}-\mu_{2}|$ [Fig. 4(a)]. The determinant of the covariance matrix |Σ| from the fitted Gaussian distribution provides a measure of the “spread” of the distribution. The difference in spread of the phasor distributions of the two regions was thus estimated as: $\Delta \Sigma =|\Sigma_{1}-\Sigma_{2}|$ [Fig. 4(b)]. The “angle” θ between major axes of the phasor distributions of the two regions was estimated as the acute angle between the eigenvectors of maximum variance of the multivariate Gaussian distributions (Fig. 4(b)). Finally, the “symmetry” of the Gaussian distribution can be quantified as the ratio of the variances along the orthogonal directions, $s=\frac{\sigma_{p}^{2}}{\sigma_{q}^{2}}$ [Fig. 4(c)]. The difference in symmetry of the phasor distributions of the two regions was thus estimated as: $\Delta s=|s_{1}-s_{2}|$.

The fluorescence frequency response H(p,q,ω) is bandlimited with a bandwidth of ∼60  MHz. To cover the bandwidth of H(p,q,ω), only the first nine frequency components of H(p,q,ω) were selected, corresponding to the frequencies 5.6, 11.2, 16.8, 22.4, 28, 33.6, 39.2, 44.8, and 50.4 MHz. These frequency values are the first nine harmonics of the signal Fourier spectrum, which was calculated at a frequency resolution of 5.6 MHz (sampling frequency/#samples=2.5  GHz/(3×149)). For each of these frequency components, the four phasor features were computed, resulting in a total of 36 phasor features.

### 2.4 Feature Selection

**Fig. 5:**
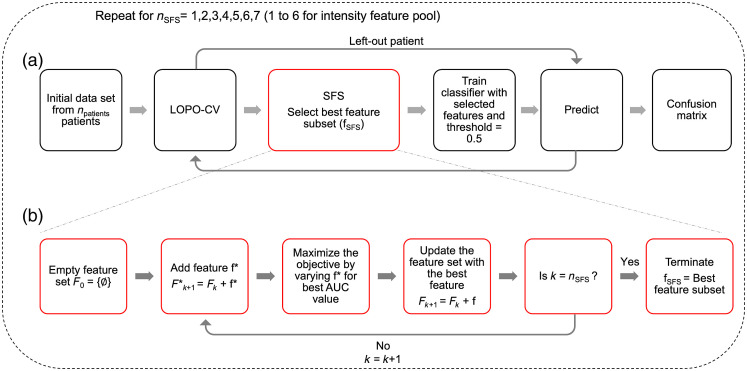
Flow diagram showing (a) feature selection process using LOPO-CV along with SFS algorithm, and (b) detailed steps involved in the SFS algorithm. The number of features selected, $n_{\mathrm{SFS}}$, is varied from 1 to 7 for all feature pools and 1 to 6 for intensity feature pool. SFS, sequential forward search; LOPO-CV, leave-one-patient-out cross-validation; AUC,– area under the curve.

For maFLIM dermoscopy-based automated classification of benign versus malignant skin lesions, a simple quadratic discriminant analysis (QDA) model was explored with the global features described in Sec. 2.3. Three different pools of global features were considered: (1) intensity ($n_{\text{features}}=6$), (2) biexponential ($n_{\text{features}}=12$), and (3) phasor ($n_{\text{features}}=36$) features, where $n_{\text{features}}$ is the number of features in each feature pool. In addition to these individual feature pools, different combinations of the feature pools were also considered: phasor ∪ biexponential ($n_{\text{features}}=48$), phasor ∪ intensity ($n_{\text{features}}=42$), intensity ∪ biexponential ($n_{\text{features}}=18$), and phasor ∪ biexponential ∪ intensity ($n_{\text{features}}=54$). Feature selection using sequential forward search (SFS)^47^ was performed on each feature pool independently using a leave-one-patient-out cross-validation (LOPO-CV) strategy, whereby the data of one patient are left out at each fold [Fig. 5(a)]. This assures that the left-out patient data are not used for feature selection and model training. Unlike exhaustive search where every possible combination of features is examined, SFS is computationally simpler and provides an efficient strategy to investigate the importance of the available features. The steps involved in the SFS feature selection process are shown in Fig. 5(b). The feature subset yielding the highest area under the curve (AUC) value in receiver operator characteristics (ROC) analysis was selected ($f_{\mathrm{SFS}}$) at each iteration of the LOPO-CV. Subsequently, the QDA classifier is retrained using $f_{\mathrm{SFS}}$ at each LOPO-CV iteration and then tested using the left-out patient data. A threshold of 0.5 on the generated posterior probability classifies the left-out patient data into either benign or malignant. Upon accomplishing all folds, each patient data had been used as test, and a confusion matrix was constructed to estimate the classification model performance. The number of features selected during SFS ($n_{\mathrm{SFS}}$) is kept constant in each fold of the LOPO-CV. Therefore, to determine the number of features that can produce the best classifier, the experiments are repeated for different values of $n_{\mathrm{SFS}}$, varying from 1 to 7 (1 to 6 for intensity feature pool). The maximum number of features in the feature set is chosen based on Hua et al.,^48^ where the optimal number of features for feature sets with some degree of correlation is $\sqrt{n_{\mathrm{data}}}$, where $n_{\mathrm{data}}$ is the number of data points. In this case, the maximum number of features is chosen as the closest and lower integer value to $\sqrt{n_{\text{lesions}}}=\sqrt{60}=7.75∼7$. The value of $n_{\mathrm{SFS}}$ that produced the best F-score computed from the final confusion matrix was then chosen as the number of features in the final feature set ($n_{\text{selected}}$). This is because F-score gives a combined estimate of both the sensitivity and specificity. However, in cases when two values of $n_{\mathrm{SFS}}$ produce the same F-score, a higher sensitivity is given more preference. This is because it is critical to ensure that the malignant lesions are correctly classified to provide adequate and timely treatment to the patients.

Since the LOPO-CV iterates $N_{\text{patient}}$ times, where $N_{\text{patient}}$ is the number of patient data, the features $f_{\mathrm{SFS}}$ selected in each iteration for a particular value of $n_{\mathrm{SFS}}$ depend on the ($N_{\text{patient}}-1$) patient data that are not left out by that iteration. Thus, there can be some variation in the features that are picked out in each iteration of the LOPO-CV. Therefore, the selection frequency is noted, which is defined as the number of times each feature becomes part of the $f_{\mathrm{SFS}}$ during all iterations of the LOPO-CV. This allowed to identify the most frequent (thus most relevant) features ($f_{\text{selected}}$) from each feature pool. It is to be noted that the number of features in each $f_{\text{selected}}$ is denoted as $n_{\text{selected}}$.

### 2.5 Classification of Skin Cancer Lesions Using Selected Features

**Fig. 6:**
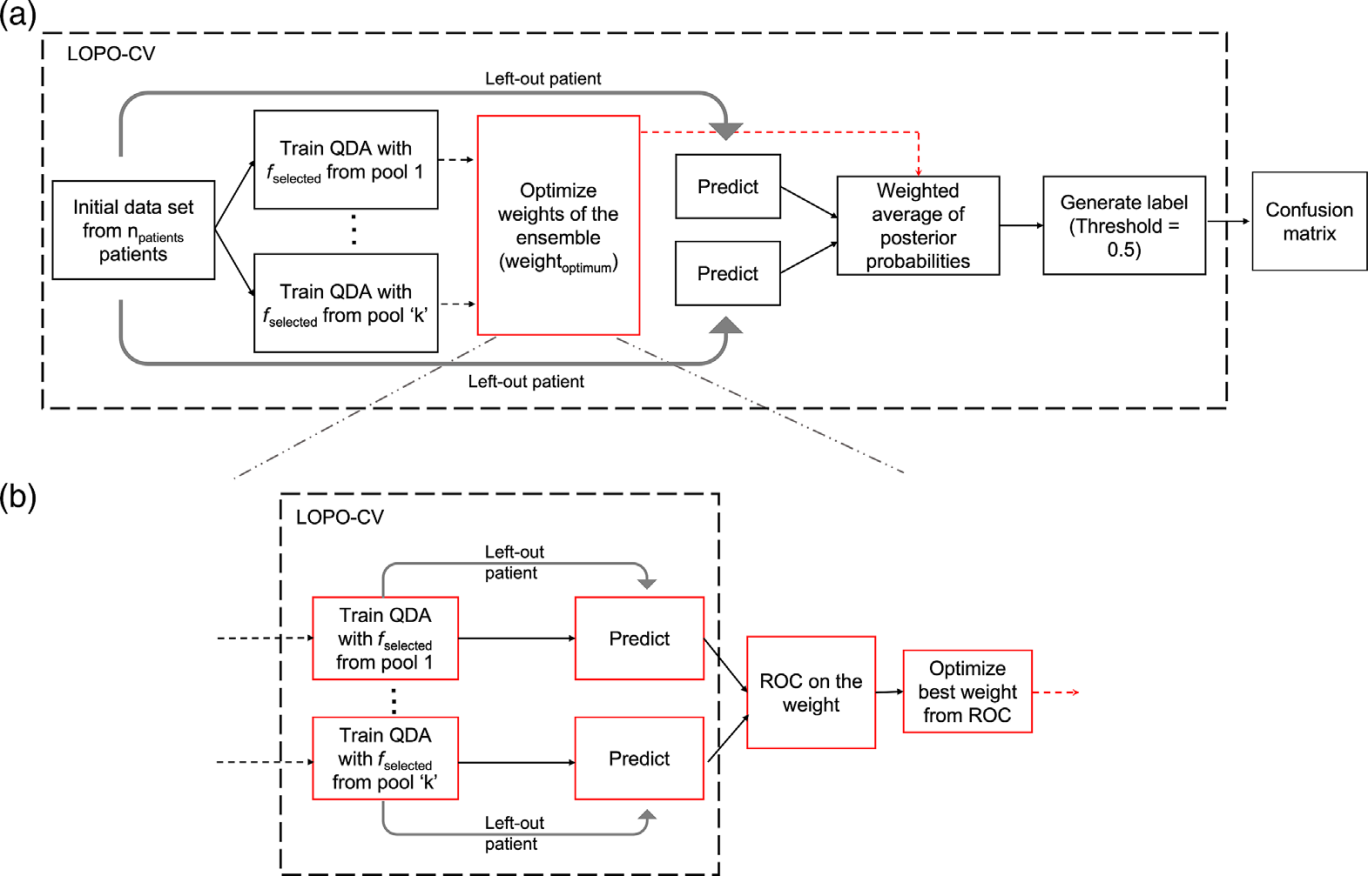
(a) Schematic of classification of skin lesions. The posterior probabilities from the individual classifiers are combined in an ensemble fashion. (b) Weight optimization for the ensemble classifier. The optimum weight is selected from the ROC curve. LOPO-CV, leave-one-patient-out cross-validation; QDA, quadratic discriminant analysis; ROC, receiver operator characteristics.

QDA classifiers trained on the best features, $f_{\text{selected}}$, from the three feature pools as explained in Sec. 2.4 were also combined in an ensemble fashion as shown in Fig. 6(a). Separate classifiers are trained on “k” feature pools with an LOPO-CV loop. If all the feature pools are used, k=3, otherwise, k=2. The left-out patient data from the LOPO-CV are tested on each of the individual classifiers, generating a set of posterior probabilities, $P_{\text{pool-}1},P_{\text{pool-}2},\ldots ,P_{\text{pool-}k}$, corresponding to classifiers trained on each feature pool. Here, Pool-k is either phasor, intensity, or biexponential feature pool. Subsequently, a weighted average of the posterior probabilities is computed as

$$P=w_{1}P_{\text{pool}-1}+\;w_{2}P_{\text{pool}-2}\ldots +w_{k}P_{\text{pool}-k},$$ (18)

$$w_{1}+w_{2}+\ldots +w_{k}=1,$$ (19)

where $w_{1},w_{2},\ldots ,w_{k}$ are the weights on the posterior probabilities generated from each feature pool, while the sum of weights equals one. A threshold of 0.5 on the weighted average of the posterior probabilities assigns a label for the left-out patient data. Figure 6(b) shows the process of weight optimization for the ensemble classifiers. Weight optimization is performed within another LOPO-CV loop. The classifiers are trained on the data from $\left(n_{\text{patients}}-2\right)$ patients and the left-out patient data are predicted to generate corresponding posterior probabilities. These posterior probabilities are combined using a weighted average. The weights are varied from 0 to 1 in steps of 0.1. The corresponding sensitivities and specificities are obtained using a threshold of 0.5 on the weighted-average probability. An ROC curve is constructed on these weights, and the weight closest to the ideal point ([0,1]) is selected.

## 3 Results

### 3.1 maFLIM Dermoscopy Clinical Imaging of Skin Lesions

The distribution of patients ($n_{\text{patients}}=30$) and lesions ($n_{\text{lesions}}=60$) imaged in this study showing benign and malignant conditions is provided in Table 3. Benign lesions included solar lentigo and pSK, while malignant lesions included pigmented superficial BCC, pigmented nodular BCC, and melanoma.

**Table 3:** Distribution of imaged benign and malignant lesions.

	Type	No. patients	No. lesions
Benign	Solar lentigo	2	10
	Pigmented seborrheic keratosis	15	31
Malignant	Pigmented superficial BCC	2	6
	Pigmented nodular BCC	5	5
	Melanoma	6	8

**Fig. 7:**
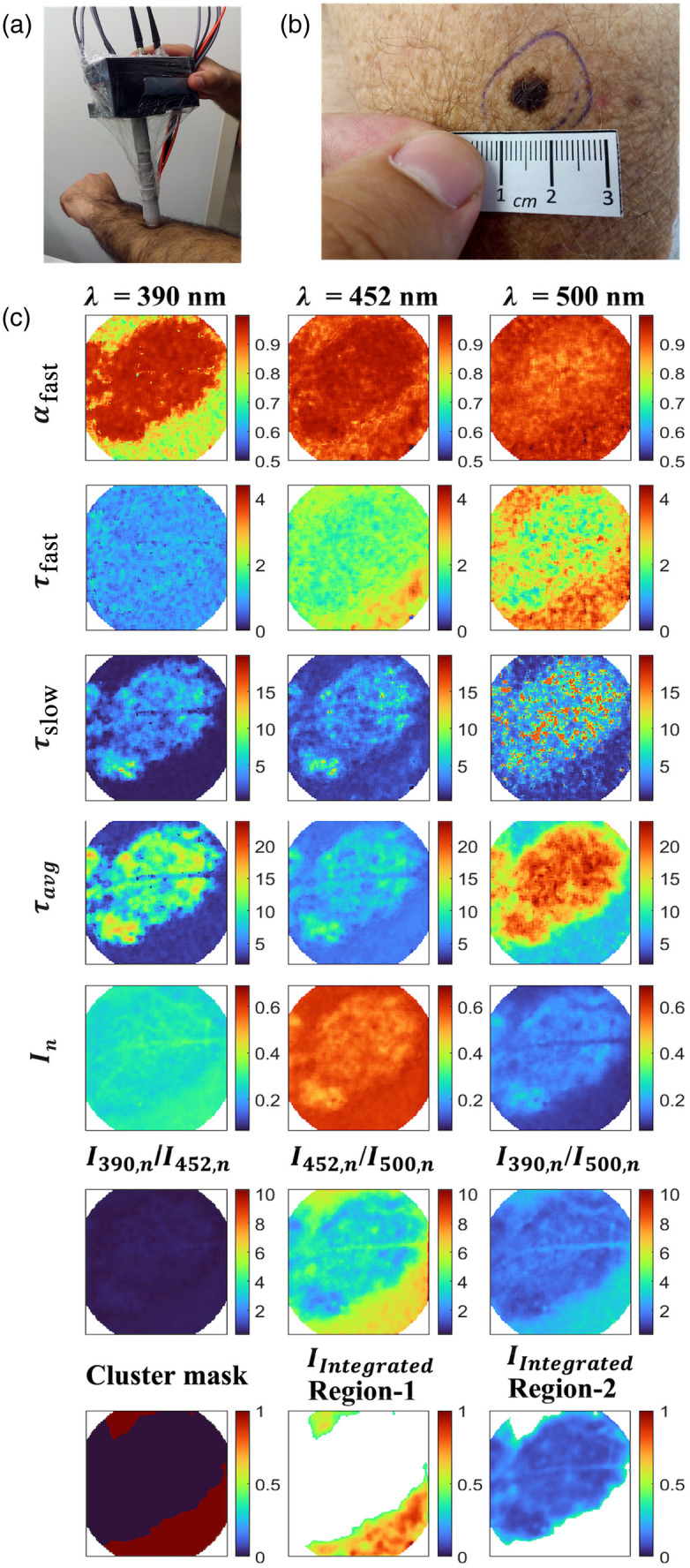
(a) Handheld maFLIM dermoscope imaging the forearm of a patient. (b) Clinical photograph of a melanoma lesion. (c) Time-domain maFLIM feature maps of a melanoma lesion. The columns show the feature maps corresponding to the three emission channels. First row shows the weight of the fast decay. Second row shows the fast lifetime maps, while the third row shows the slow lifetime maps. Average lifetime maps are shown in the fourth row. Fifth row shows the integrated intensity maps of each spectral emission channel, and the ratio of the intensities are shown in the sixth row. The last row shows the cluster mask generated for the lesion and the integrated intensities from all the channels for the clustered regions 1 and 2. The horizontal strip in the images is due to the presence of hair on the skin during imaging.

Figure 7(a) shows a handheld maFLIM dermoscope imaging the forearm of a patient. The clinical photograph of a sample melanoma skin lesion is shown in Fig. 7(b), and its corresponding maFLIM feature maps are shown in Fig. 7(c). The scales of the feature maps across the three spectral wavelengths are kept the same for comparison purposes. Most of the feature maps (including $\alpha_{\text{fast},390}$, $\alpha_{\text{fast},452}$, $\tau_{\text{fast},452}$, $\tau_{\text{fast},500}$, $\tau_{\text{slow},390}$, $\tau_{\text{slow},452}$, $\tau_{\text{slow},500}$, $\tau_{\mathrm{avg},390}$, $\tau_{\mathrm{avg},452}$ $\tau_{\mathrm{avg},500}$, $I_{n,452}$, $I_{n,500}$, $I_{452,n}/I_{500,n}$ and $I_{390,n}/I_{500,n}$) clearly show two distinguishable regions: the center and the surrounding regions. The cluster masks for this sample image are also shown in the last row of Fig. 7(c). The maps showing the two clustered regions: region-1 and region-2 are plotted using the normalized integrated intensities ($I_{\text{Integrated}}$) from the three spectral emission channels.

The relative contributions of fast lifetime ($\alpha_{\text{fast}}$) for the spectral channels 390 and 452 nm exhibit a higher value in the central region compared with the surrounding regions. Fast lifetime ($\tau_{\text{fast}}$) values for the spectral channels 452 and 500 nm are lower in the central region than the surrounding. Slow lifetime ($\tau_{\text{slow}}$) values for all the three spectral channels are higher in the center than the surrounding regions. The average lifetimes ($\tau_{\mathrm{avg}}$) of the pixels from the central region are higher than the surrounding parts. The relative intensity ($I_{n}$) values for spectral channel 452 nm is lower in the center compared to the surrounding, while that for the spectral channel 500 nm is higher in the center compared with the surrounding. The ratios of the intensities, $I_{452,n}/I_{500,n}$ and $I_{390,n}/I_{500,n}$, are lower in the central region compared with the surrounding regions.

### 3.2 Feature Selection

As explained in the feature selection process in Sec. 2.4, the multiple lesions from a single patient constitute the left-out data in each iteration; thus, the number of lesions that are tested in each iteration varies depending on the number of lesions that are imaged for the left-out patient. In this way, every patient becomes part of the testing, and a confusion matrix is generated after all LOPO-CV iterations are completed. As mentioned in Sec. 2.4, the number of features $n_{\mathrm{SFS}}$ was varied from 1 to 6 for intensity feature pool, and 1 to 7 for phasor and biexponential feature pools. The optimum number of features $n_{\text{selected}}$ is chosen based on the highest F-score. Table 4 shows values of accuracy, sensitivity (Sn), specificity (Sp), and F-score obtained when classifying benign and malignant skin lesions trained individually on the three feature pools (phasor, biexponential, and intensity). The table shows the results for $n_{\text{selected}}$ number of features in each feature pool. Results obtained during feature selection for all the values of $n_{\mathrm{SFS}}$ is given in Tables S1–S3 in the Supplemental Material. QDA models trained on five biexponential features yielded the best performance with 75% accuracy, 84.21% sensitivity, 70.73% specificity, and 68.09% F-score. QDA models trained on six phasor features yielded the next best performance with 76.67% accuracy, 68.42% sensitivity, 80.49% specificity, and 65% F-score. QDA models trained on the intensity feature pool resulted in 84.21% sensitivity, but only 31.71% specificity.

**Table 4:** Performance metrics and confusion matrices obtained during feature selection with phasor, biexponential, and intensity feature pools.

Feature pool (total no. of features)	$n_{\text{Selected}}$	Accuracy (%)	Sn (%)	Sp (%)	F score (%)	Confusion matrices			
						True	Predicted		
							Benign	Malignant	
Phasor (36)	6	76.67	68.42	80.49	65.00	Benign	33	8	
						Malignant	6	13	
Bi-exponential (12)	5	75.00	84.21	70.73	68.09	Benign	29	12	
						Malignant	3	16	
Intensity (6)	1	48.33	84.21	31.71	50.79	Benign	13	28	
						Malignant	3	16	
Phasor ∪ biexponential (48)	4	56.67	63.17	56.10	48.98	Benign	23	18	
						Malignant	7	12	
Phasor ∪ intensity (42)	6	53.33	63.16	48.79	46.15	Benign	20	21	
						Malignant	7	12	
Biexponential ∪ intensity (18)	7	63.33	63.16	63.41	52.17	Benign	26	15	
						Malignant	7	12	
Phasor ∪ biexponential ∪ intensity (54)	6	61.67	47.37	68.29	43.90	Benign	28	13	
						Malignant	10	9	

The results from the combined feature pools show poor performance with low sensitivities and specificities. This is because the features selected by the SFS algorithm in the earlier iterations may not be the best when combined with those selected in the later iterations. From these results, it can be inferred that the five biexponential features and the six phasor features have potential in classifying benign and malignant skin lesions. The confusion matrices of the classifiers are also shown in Table 4.

### 3.3 Feature Relevance

To identify the important features in each feature pool, the number of times each feature becomes a part of $f_{\mathrm{SFS}}$ during all iterations of the LOPO-CV was recorded. If a feature is selected at least 50% times during all the iterations, it will be considered an important feature and added to the $f_{\text{selected}}$ of that feature pool. Table 5 lists the important features from each feature pool and their selection frequencies during LOPO-CV. Thus, we can summarize the selected features from each feature pool as

$$(f_{\text{selected}}{)}_{\text{phasor}}=[{\text{symmetry}}_{16.8\;\mathrm{MHz}},{\text{symmetry}}_{33.6\;\mathrm{MHz}},{\text{symmetry}}_{39.2\;\mathrm{MHz}}{\text{symmetry}}_{50.4\;\mathrm{MHz}},{\text{spread}}_{33.6\;\mathrm{MHz}},{\text{distance}}_{50.4\;\mathrm{MHz}}]\;(f_{\text{selected}}{)}_{\mathrm{biexponential}}=[\alpha_{\text{fast},390},\tau_{\text{slow},390},\alpha_{\text{fast},452},\tau_{\text{fast},452},\;\alpha_{\text{fast},500}]\;{\left(f_{\text{selected}}\right)}_{\text{intensity}}=[I_{452,n}].$$

**Table 5:** Summary of important features selected from each feature pool along with their ranks.

Feature pool	$f_{\text{selected}}$	Selection frequency percentage (%)
Phasor	Symmetry at 39.2 MHz	93.3
	Spread at 33.6 MHz	90.0
	Symmetry at 16.8 MHz	90.0
	Symmetry at 50.4 MHz	86.7
	Symmetry at 33.6 MHz	56.7
	Distance at 50.4 MHz	50.0
Biexponential	$\tau_{\text{fast},452}$	96.7
	$\tau_{\text{slow},390}$	93.3
	$\alpha_{\text{fast},452}$	80.0
	$\alpha_{\text{fast},390}$	76.7
	$\alpha_{\text{fast},500}$	50.0
Intensity	$I_{452,n}$	96.7

### 3.4 Classification of Skin Cancer Lesions Using Selected Features

The methodology of classifying skin lesions using the selected features is explained in Sec. 2.5. As shown in Fig. 6, “k” QDA classifiers trained separately on the $f_{\text{selected}}$ features from “k” feature pools are combined in an ensemble fashion. Four different combinations of feature pools are used for constructing ensemble classifiers, as shown in Table 6. A weighted average of the posterior probabilities generated from the “k” QDA classifiers is calculated to predict the label of the left-out patient data. Since the weights are optimized within an LOPO-CV loop, there can be some variation in the weights selected during the $n_{\text{patients}}$ iterations.

**Table 6:** Performance metrics of ensemble classifiers trained with multiple combinations of feature pools.

Feature sets for ensemble	Accuracy (%)	Sn (%)	Sp (%)	F-score (%)	Confusion matrices		
					True	Predicted	
						Benign	Malignant
${\left(f_{\text{selected}}\right)}_{\text{biexponential}}+{\left(f_{\text{selected}}\right)}_{\text{intensity}}$	71.67	84.21	65.85	65.31	Benign	27	14
					Malignant	3	16
${\left(f_{\text{selected}}\right)}_{\text{phasor}}+{\left(f_{\text{selected}}\right)}_{\text{biexponential}}$	88.33	84.21	90.24	82.05	Benign	37	4
					Malignant	3	16
${\left(f_{\text{selected}}\right)}_{\text{phasor}}+{\left(f_{\text{selected}}\right)}_{\text{intensity}}$	86.67	78.95	90.24	78.95	Benign	37	4
					Malignant	4	15
${\left(f_{\text{selected}}\right)}_{\text{phasor}}+{\left(f_{\text{selected}}\right)}_{\text{biexponential}}+{\left(f_{\text{selected}}\right)}_{\text{intensity}}$	88.33	84.21	90.24	82.05	Benign	37	4
					Malignant	3	16

**Fig. 8:**
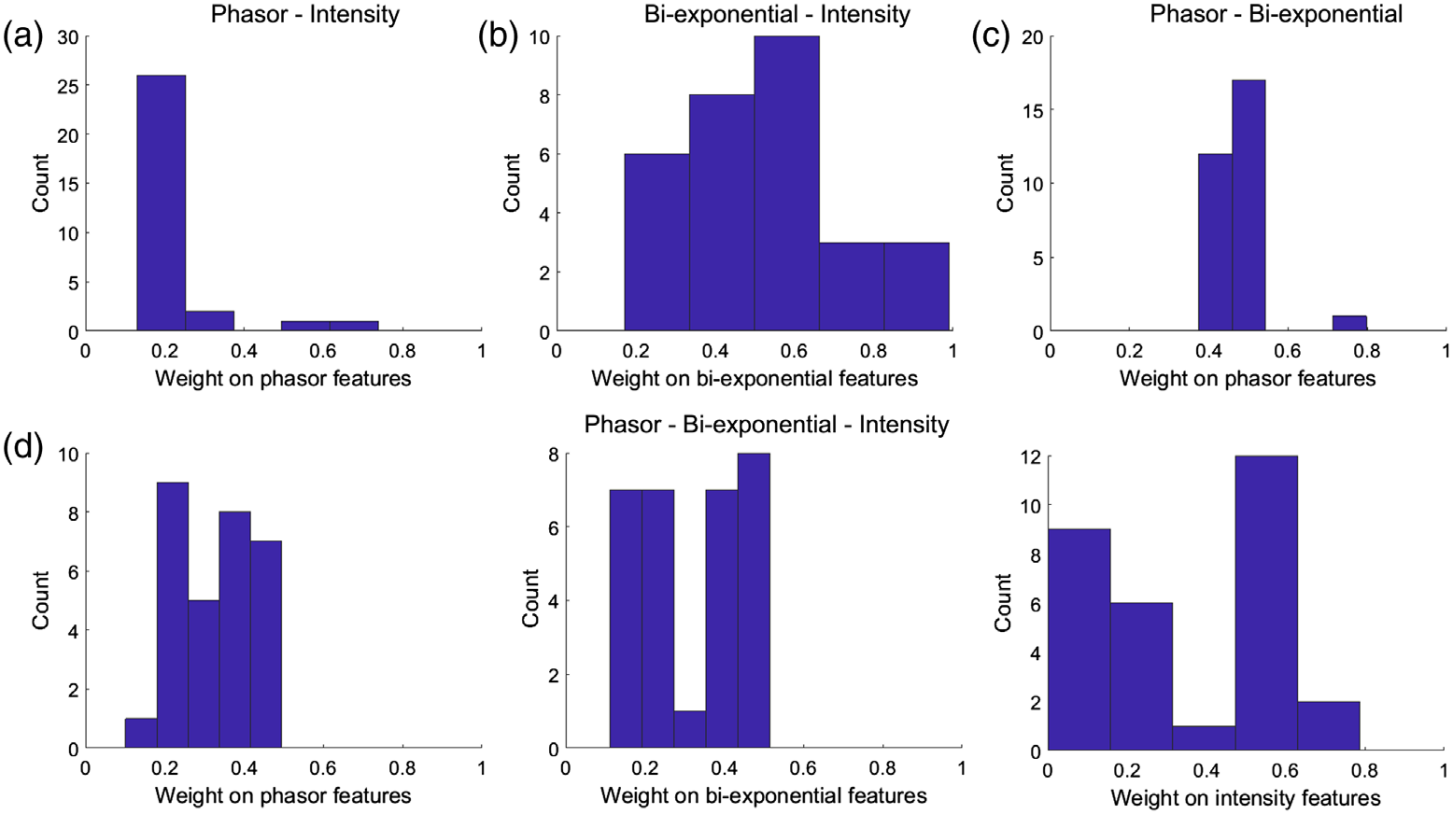
Histogram of weights on one of the feature pools, when combined in an ensemble fashion for (a) phasor-intensity, (b) biexponential-intensity, (c) phasor-biexponential, and (d) phasor-biexponential-intensity feature pools.

Figure 8 shows the histograms of the weights obtained during all the iterations for the ensemble classifiers using the four feature pool combinations. Since the sum of weights of “k” feature pools is one, it is sufficient to show the weights of (k-1) feature pools. It can be seen from Table 6 that the ensemble combination of QDA classifiers trained on phasor and biexponential features as well as the ensemble combination of all the three feature pools, produced the best performance with 88.33% accuracy, 84.21% sensitivity, 90.24% specificity, and F-score of 82.05%. The next best performance is obtained by the ensemble combination of phasor and intensity feature pools with an accuracy of 86.67%, sensitivity of 78.95, specificity of 90.4%, and an F-score of 78.95%. Ensemble combination of intensity and biexponential features produced an accuracy of 71.67%, sensitivity of 84.21%, specificity of 65.85%, and F-score of 65.31%. Table 6 also shows the confusion matrices for all the classifiers. While analyzing the weights on the feature pools during the ensemble combination, it can be seen in Figs. 8(a) and 8(b) that intensity features have a higher weightage when combined with phasor features. When phasor and biexponential features are combined, both the feature pools have similar weightage as shown in Fig. 8(c). Similarly, when biexponential and intensity feature pools are combined, both the pools have comparable weightage. When all the feature pools are combined, it can be seen from Fig. 8(d) that the phasor and biexponential features pools have similar weight distribution in the range [0.1, 0.5]. The weights on the intensity feature pool are widely dispersed in the range [0, 0.8].

## 4 Discussion

In this study, clinical widefield autofluorescence imaging of benign and malignant pigmented skin lesions was successfully performed in 30 patients using a recently developed maFLIM dermoscope.^40^ The resulting maFLIM images from 60 pigmented lesions enabled exploring steady-state (intensity) and time-resolved (biexponential, phasor) autofluorescence global features. Results based on rigorous cross-validation methods demonstrate that simple ML classification models (QDA) based on selected time-resolved autofluorescence global features have the potential to provide discrimination of malignant from benign pigmented skin lesions.

To the best of our knowledge, only one published work has reported the use of machine learning models based on autofluorescence lifetime imaging features for the classification of pigmented skin lesions.^12^ In that study, however, only skin melanoma lesions were imaged, and the classification task was restricted to discriminate early-stage from advanced-stage skin melanoma. In contrast, a more comprehensive set of pigmented skin lesions were imaged in this work (two benign and three malignant lesion categories). Moreover, the classification task focused on discriminating malignant from benign pigmented lesions, which might be clinically more relevant for early detection of skin cancer.

In multidimensional imaging data, such as in maFLIM, image features can be extracted at the pixel or the image level. We have recently explored pixel-level maFLIM features for the classification of oral dysplasia and early-stage cancer.^49^ Pixel-level features, however, require the labeling of each pixel which is generally impractical. In this work, the maFLIM data were labeled at the lesion level based on the histopathology diagnosis obtained from the lesion biopsy samples; therefore, an image-level global feature extraction strategy was preferred. As shown in Fig. 7, two regions were frequently observed in the maFLIM images, corresponding to pixels either within or outside the lesion extension. In an attempt to reduce interpatient variability, the explored relative features were defined in terms of difference in autofluorescence properties between the two regions identified in each lesion maFLIM image. This strategy of using global (image-level) and relative features can find applications in many other classification tasks based on optical imaging data.

The performance of ML classification models needs to be carefully estimated when trained on limited sample size. To minimize overfitting and avoid overoptimistic performance estimations, a rigorous strategy was adopted for feature selection, model training, and performance estimation. First, the maximum number of features allowed (seven) was limited based on the sample size.^48^ Second, a simple nonlinear classification model (QDA) was adopted. Third, cross-validation was applied at the patient-level (LOPO-CV) to ensure that data from the same patient is not used for both training and validation. Fourth, feature selection was performed together with model training to make sure that the validation data are not used during neither feature selection nor model training. It should be noted that at each fold of the cross-validation strategy, a different classification model (with different selected features and model parameters) is applied to the validation set. Thus, although a single optimal model is not necessarily defined, this approach still enables identifying relevant features and providing unbiased classification performance estimation.

Classification performance was dependent of the feature pool used in the model. The most frequently selected intensity feature was $I_{452,n}$ which is associated to NADH fluorescence contribution. Although the classification models using intensity features showed good sensitivity (∼84%), their specificity was poor (∼31%). This means that steady state intensity features are not sufficient to minimize false positives while discriminating benign and malignant lesions. Classification models using biexponential features showed similar sensitivity (∼84%) than those with intensity features, but significantly higher specificity (∼70%). These results indicate that time-resolved properties of pigmented skin lesion autofluorescence could represent biomarkers of skin cancer. While examining the selected features from the biexponential feature pool, it can be seen from Table 5 that the most frequently selected features were associated to NADH ($\tau_{\text{fast},452}$, $\alpha_{\text{fast},452}$) and collagen ($\tau_{\text{slow},390}$, $\alpha_{\text{fast},390}$) fluorescence temporal dynamics. The fast lifetime component of NADH ($\tau_{\text{fast},452}$, $\alpha_{\text{fast},452}$) is associated with the free state of the molecule,^19^ suggesting that metabolic pathway changes induced by malignant transformations alter the microenvironment of NADH molecules. Unlike NADH or FAD, the collagen is not involved in cellular respiration and is not part of metabolic pathways. Changes in collagen autofluorescence response can occur in benign and malignant lesions as they mostly correspond to skin thickening, extracellular matrix remodeling, and texture changes.^23^ Therefore, the selected features corresponding to collagen autofluorescence ($\tau_{\text{slow},390}$, $\alpha_{\text{fast},390}$) can provide high sensitivity but may not contribute to high specificity leading to several false positives as can be seen in Table 4.

The phasor representation of fluorescence lifetime imaging data analysis is a noniterative, model-free, fast approach to visualize the lifetime components (and their distributions) of the fluorescence emission of a sample.^50^^–^^53^ In this work, a modified version of this method was applied to the clinical multispectral maFLIM data, and a set of global image features were extracted from the corresponding phasor representation of the two regions present in each lesion maFLIM image (Fig. 4). The processing times taken for computing phasor and biexponential features from one sample lesion using a computer with an i7 Intel core processor and 48 GB RAM were found to be 10.5 and 251.8 s, respectively. Therefore, phasor-based features are simpler and ∼25 times faster than traditional time resolved biexponential features. Unlike biexponential maFLIM features, the phasor features explored cannot be directly interpreted in terms of the skin autofluorescent constituents. Nevertheless, the classification models using phasor features showed superior specificity (∼80%) than those using biexponential features, suggesting that these two different pools of time-resolved maFLIM features might be complementary. Phasor-based features are computed from the concatenated signal and contain information from all three emission channels. It is challenging to develop solid reasoning for the high specificity obtained from these features; however, since every phasor-based feature contains information from all three channels, each channel’s contribution is not quantifiable.

Feature selection starting with combinations of feature pools were also explored, although the resulting models showed lower classification performance overall (Table 4). On the other hand, ensemble classifiers based on models using the most frequently selected features of each pool outperformed any other models (Table 5). In particular, the ensemble classifier combining the models based on the most frequently selected biexponential and phasor features resulted in the best performance overall (∼84% sensitivity and ∼90% specificity). Moreover, the optimum weights identified for these ensemble models indicate that both the phasor and biexponential features contribute equally to the weighted probability [Fig. 8(c)]. These results further indicate that these two maFLIM feature pools might be complementary, as biexponential features seem to contribute to higher sensitivity, while phasor features to higher specificity.

Some of the studies summarized in Table 1 produced superior classification performances compared with that reported in this work. However, those studies employed complex machine learning and deep learning algorithms, such as Adaboost,^10^ BoF,^10^ and CNN’s for classification,^18^ and feature extraction.^17^ This work uses a simple QDA machine learning model to analyze the performance with different feature pools. It is plausible that more complex machine learning classification models, such as neural networks, might provide superior performance. Given the limited data available for training and validation, however, a simple QDA model was selected, as it still enables nonlinear decision boundaries while reducing the chances of overfitting. In addition, by choosing a simple classification method, it is possible to analyze other aspects of classification model, such as the feature pools, number of features, and ensemble combinations of the feature pools.

Recent advances in artificial intelligence (AI) are allowing the development of CAD systems for discriminating benign from malignant skin lesions based on digital dermoscopy data. Although these CAD systems have not been translated yet to the clinic, preliminary validation studies demonstrate their potential to discriminate typical benign from malignant lesions. Dermoscopy data, however, provide information limited to the visual appearance of the lesions; thus, AI-assisted digital dermoscopy is less suitable for the discrimination of visually similar benign and malignant skin lesions. maFLIM dermoscopy data, on the other hand, can capture autofluorescence-based biochemical and metabolic biomarkers of skin malignant transformation, which is independent of lesion visual appearance. This property is especially important in distinguishing visually similar benign and malignant lesions, thus minimizing the number of false positives and, in turn, reducing the number of unnecessary biopsies. In particular, the lesion types included in this work—pSK and melanoma, are visually similar benign and malignant lesions, which can be easily misdiagnosed during dermoscopic evaluation. Therefore, maFLIM dermoscopy has the potential to complement digital dermoscopy by providing superior performance in the discrimination of visually similar benign and malignant skin lesions.

### 4.1 Study Limitations

Although this preliminary clinical study demonstrates the potential of maFLIM-derived autofluorescence features to discriminate malignant from benign pigmented skin lesions, a number of limitations are recognized. First, the database of maFLIM images is limited in both the type of benign and malignant skin conditions, and the number of samples per condition. A more comprehensive and larger database will be needed to fully develop accurate enough classification methods for skin lesion discrimination and to rigorously quantify their performance in prospective studies. Second, the lack of histopathology-based assessment of the maFLIM imaging data at the pixel-level prevented to specifically quantify the capabilities of maFLIM dermoscopy as a tool for not only detecting malignant skin lesions but also determining their true extension and margins. Third, the current maFLIM dermoscopy system provides nonspecific excitation and spectral detection of skin autofluorescence component emission. Finally, the current implementation of the ML classification models does not allow for real-time processing of maFLIM data. Ongoing research efforts aiming to overcome these limitations include collecting maFLIM dermoscopy images from a plurality of nonpigmented and pigmented skin lesions from patients of various skin tones, performing accurate pixel-level registration between the lesion maFLIM imaging data and histopathology tissue sections, developing improved maFLIM dermoscope systems with multiwavelength excitation and narrow-band emission detection capabilities, and implementing optimized CAD using field programmable gate arrays and graphics processing units technologies for real-time maFLIM data processing, pixel-level classification, and tissue mapping visualization.

### 4.2 Clinical Perspective

The incidence of skin cancer including melanoma continues to increase yet most providers is forced to rely on their own visual recognition skills and experience to identify concerning lesions. In addition, many patients do not have access to a trained dermatologist which can place them in a potentially precarious situation since early detection of skin cancer leads to better survival rates. The importance of early detection cannot be understated, as it not only saves lives but also reduces the invasiveness of the treatment patients undergo and conserves precious medical resources, leading to quality, cost-conscious care. A noninvasive, label-free, fast, accurate, and objective tool capable of discriminating most common malignant from benign skin lesions would improve the clinical management of patients. Currently, there is no objective device providers can use to independently identify cancerous skin lesions. In the hand of primary care physicians, such a tool will enable the early identification of patients in need of referral to a dermatologist. In addition, the dermatologists could use such tool to identify lesions in need of a biopsy, thus reducing the rate of unnecessary biopsies and adverse events such as pain, infection, and scarring. Such a tool would also assist with monitoring cancer recurrence without the need of regular and frequent biopsies. This work demonstrates that maFLIM dermoscopy aided by ML models could potentially have the capabilities of such tool, thus impacting the clinical management of skin cancer patients for the better.

## 5 Conclusion

The results of this study demonstrate the capabilities of maFLIM dermoscopy to clinically image a plurality of autofluorescence biomarkers of malignant skin pigmented lesions. Moreover, some of these autofluorescence biomarkers were identified as promising for malignant lesion identification, particularly those quantifying the time-resolved fluorescence characteristics of skin lesions. In addition, these relevant autofluorescence biomarkers were successfully used as features in ML models trained to discriminate malignant from benign pigmented skin lesions with promising accuracy (∼84% sensitivity and ∼90% specificity). Further developments in maFLIM instrumentation and image analysis methods could result in clinical tools for noninvasive, label-free, accurate, and objective *in situ* detection of malignant from benign skin lesions, with the potential to impact the clinical management of skin cancer patients.
