# Supplementary material for: Discrimination of cancerous from benign pigmented skin lesions based on multispectral autofluorescence lifetime imaging dermoscopy and machine learning
Source: J Biomed Opt. 2022 Jun 14;27(6):066002. doi: 10.1117/1.JBO.27.6.066002 (PMC9196925; doi:10.1117/1.JBO.27.6.066002)
Supplement: Supplementary file 1 [file JBO_027_066002_SD001.pdf]

# Supplemental Material

# Discrimination of cancerous from benign pigmented skin lesions based on multispectral autofluorescence lifetime imaging dermoscopy and machine learning

**Priyanka Vasanthakumari,[a] Renan A. Romano,[b] Ramon G. T. Rosa,[b] Ana G. Salvio[c], Vladislav Yakovlev[a], Cristina Kurachi[b], Jason M. Hirshburg[d], Javier A. Jo[e,*]**

[a] Texas A&M University, Department of Biomedical Engineering, College Station, USA
[b] University of São Paulo, São Carlos Institute of Physics, São Paulo, Brazil
[c] Skin Department of Amaral Carvalho Hospital, São Paulo, Brazil
[d] University of Oklahoma Health Science Center, Department of Dermatology, Oklahoma City, OK, USA
[e] University of Oklahoma, School of Electrical and Computer Engineering, Norman, USA

The feature selection procedure is explained in section 2.4 of the main paper and illustrated in Fig. 5. The number of features that are selected after sequential forward search ($n_{SFS}$) is varied from 1 to 7 (1 to 6 for intensity feature pool). Table S1 shows the performance metrics obtained during feature selection for all values of $n_{SFS}$ when classifiers are trained with individual feature pools – phasor, bi-exponential and intensity. Feature selection was also performed with combination feature pools, whereby the individual feature pools were combined in pairs. Table S2 shows the performance metrics obtained using the combined feature pool for all values of $n_{SFS}$. The results of feature selection when all the three feature pools are combined are shown in Table S3. The best $n_{SFS}$ ($n_{selected}$) for each feature pool (individual or combined) is chosen based on the F-scores shown in the Tables S1, S2 and S3. Table 4 in the main paper only tabulates the performance metrics for $n_{selected}$ number of features in each feature pool.

Table S1: Performance metrics obtained during feature selection with individual feature pools - phasor, bi-exponential and intensity, for all values of $n_{SFS}$

| No. features ($n_{SFS}$) | Intensity feature pool | | | | Bi-exponential feature pool | | | | Phasor feature pool | | | |
|---|---|---|---|---|---|---|---|---|---|---|---|---|
| | Acc (%) | Sn (%) | Sp (%) | F-score (%) | Acc (%) | Sn (%) | Sp (%) | F-score (%) | Acc (%) | Sn (%) | Sp (%) | F-score (%) |
| 1 | 48.33 | 84.21 | 31.71 | 50.79 | 26.67 | 52.63 | 14.63 | 31.25 | 46.67 | 78.95 | 31.71 | 48.39 |
| 2 | 35.00 | 73.68 | 17.07 | 41.79 | 30.00 | 47.37 | 21.95 | 30.0 | 41.67 | 73.68 | 26.83 | 44.44 |
| 3 | 50.00 | 42.11 | 53.66 | 34.78 | 51.67 | 68.42 | 43.90 | 47.27 | 65.00 | 68.42 | 63.41 | 55.32 |
| 4 | 28.33 | 31.58 | 26.83 | 21.82 | 60.00 | 78.95 | 51.22 | 55.56 | 68.33 | 68.42 | 68.29 | 57.78 |
| 5 | 43.33 | 57.89 | 36.59 | 39.29 | 75.00 | 84.21 | 70.73 | 68.09 | 73.33 | 73.68 | 73.17 | 63.64 |
| 6 | 40.00 | 68.42 | 26.83 | 41.94 | 66.67 | 89.47 | 56.10 | 62.96 | 76.67 | 68.42 | 80.49 | 65.00 |
| 7 | | | | | 55.00 | 84.21 | 41.47 | 54.24 | 71.67 | 52.63 | 80.49 | 54.05 |

Acc – Accuracy; Sn – Sensitivity; Sp – Specificity

Table S2: Performance metrics obtained during feature selection by combining pairs of feature pools - phasor, bi-exponential and intensity, for all values of $n_{SFS}$

| No. features ($n_{SFS}$) | Bi-exponential - Intensity feature pools | | | | Phasor - Bi-exponential feature pools | | | | Phasor - Intensity feature pools | | | |
|---|---|---|---|---|---|---|---|---|---|---|---|---|
| | Acc (%) | Sn (%) | Sp (%) | F-score (%) | Acc (%) | Sn (%) | Sp (%) | F-score (%) | Acc (%) | Sn (%) | Sp (%) | F-score (%) |
| 1 | 45.00 | 84.21 | 26.83 | 49.23 | 38.33 | 63.16 | 26.83 | 39.34 | 40.00 | 63.16 | 29.27 | 0.400 |
| 2 | 58.33 | 21.05 | 75.61 | 24.24 | 40.00 | 78.95 | 21.95 | 45.45 | 28.33 | 63.16 | 12.20 | 35.82 |
| 3 | 51.67 | 26.32 | 63.41 | 25.64 | 51.67 | 63.16 | 46.34 | 45.28 | 41.67 | 63.16 | 31.71 | 40.68 |
| 4 | 60.00 | 36.84 | 70.73 | 36.84 | 56.67 | 63.17 | 56.10 | 48.98 | 51.67 | 63.16 | 46.37 | 45.28 |
| 5 | 65.00 | 52.63 | 70.73 | 48.78 | 56.67 | 57.89 | 56.10 | 45.83 | 48.33 | 57.89 | 43.90 | 41.51 |
| 6 | 58.33 | 47.37 | 63.41 | 41.86 | 60.00 | 57.89 | 60.98 | 47.83 | 53.33 | 63.16 | 48.79 | 46.15 |
| 7 | 63.33 | 63.16 | 63.41 | 52.17 | 60.00 | 42.11 | 68.29 | 40.00 | 53.33 | 52.63 | 53.66 | 41.67 |

Acc – Accuracy; Sn – Sensitivity; Sp – Specificity

Table S3: Performance metrics obtained during feature selection by combining all the three feature pools - phasor, bi-exponential and intensity, for all values of $n_{SFS}$

| No. features ($n_{SFS}$) | Phasor - Bi-exponential - Intensity feature pools | | | |
|---|---|---|---|---|
| | Acc (%) | Sn (%) | Sp (%) | F-score (%) |
| 1 | 40.00 | 63.16 | 29.27 | 0.400 |
| 2 | 28.33 | 63.16 | 12.20 | 35.82 |
| 3 | 41.67 | 63.16 | 31.71 | 40.68 |
| 4 | 51.67 | 63.16 | 46.37 | 45.28 |
| 5 | 48.33 | 57.89 | 43.90 | 41.51 |
| 6 | 53.33 | 63.16 | 48.79 | 46.15 |
| 7 | 53.33 | 52.63 | 53.66 | 41.67 |

Acc – Accuracy; Sn – Sensitivity; Sp – Specificity

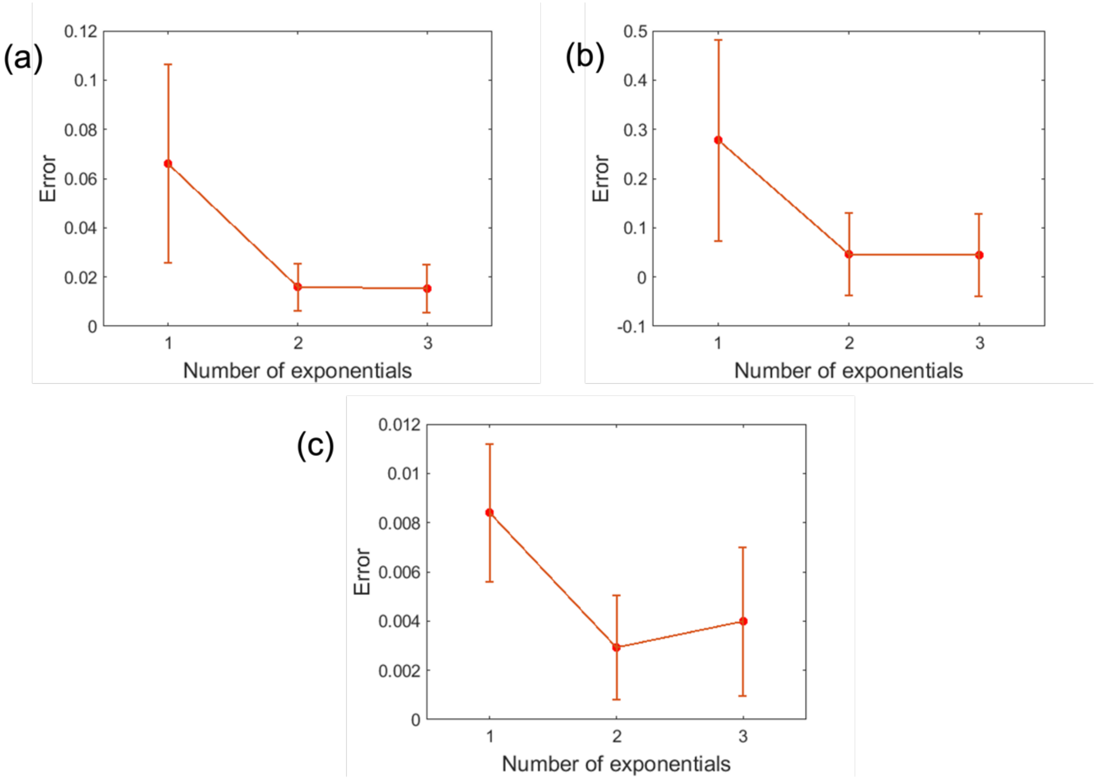


**Fig. S1** Means and standard deviations of the errors computed during fitting the fluorescence impulse response with 1, 2 and 3 exponential models for the unmasked pixels in a sample lesion maFLIM data computed for emission channels (a) 390 nm, (b) 452 nm, and (c) 500 nm
